# Supplementary material for: Longitudinal causal effect of modified creatinine index on all-cause mortality in patients with end-stage renal disease: Accounting for time-varying confounders using G-estimation
Source: PLoS One. 2022 Aug 19;17(8):e0272212. doi: 10.1371/journal.pone.0272212 (PMC9390931; doi:10.1371/journal.pone.0272212)
Supplement: Attachment — Submitted filename: Response to reviewers.docx [file pone.0272212.s002.docx]

We appreciate the academic editor and reviewers for their deep and thorough review. We have revised our manuscript in the light of their useful suggestions and comments, and we hope our revision has improved the manuscript to a level of their satisfaction.

1. Reviewer #1: The revised manuscript addressed the majority of the reviewer's comments. However, there is still a need to modify the presentation of the results. In the statement presenting the results (abstract and results section, Page 7), the direction of the association of the main effect is positive while the 95% confidence interval is negative. Please rectify this.
   "G-estimation of SAFTM denoted 43% (95% CI: -79% to -19%) shorter survival time in subjects who would have been always in decline MCI group than those who would have been always in no-decline MCI group throughout the follow-up".
   Consider using the same ratios presented in Table 2 rather than converting the ratios into percentages.

Thank you for this suggestion. We have modified the presentation of the results according to the reviewer’s comments based on Table 2.
